# Effects of genotype × environment interactions on the morphological and genetic aspects of the seed characteristics of faba beans

**DOI:** 10.1002/tpg2.70133

**Published:** 2025-10-15

**Authors:** Ahmed Sallam, Salwa M. Mahmoud, Ahmed Amro, Ameer Elfarash, Andreas Börner

**Affiliations:** ^1^ GeneBank Department, Leibniz Institute of Plant Genetics and Crop Plant Research (IPK) Stadt Seeland Germany; ^2^ Department of Genetics, Faculty of Agriculture Assiut University Assiut Egypt; ^3^ School of Biotechnology Badr University in Assiut (BUA) Assiut Egypt; ^4^ Departent of Botany & Microbiology, Faculty of Science Assiut University Assiut Egypt

## Abstract

Faba bean (*Vicia faba* L.) is one of the oldest cultivated crops in the world. It is the third most important feed grain legume globally, after soybean and lupin. It is an autogamous plant with a partial outcrossing rate ranging from 20% to 80%. The objective of most faba bean improvement programs is to enhance yield; however, yield is a complex trait influenced by many other traits. Therefore, in this study, we focused on seed traits that are related to faba bean yield. A set of 110 faba bean genotypes was tested across two different locations (Germany and Egypt) to investigate the effect of genotype–environment interactions and identify single‐nucleotide polymorphism (SNPs) related to seed characteristics. This study revealed that there is high genetic variation among genotypes in all traits in each location, and the genotype × location interactions were significant. There was a strong positive correlation among the seed characteristics within each location, but the correlations between the two locations were weak or not significant. FB‐231 and FB‐227 performed very well in both countries based on the selection index (SI) values, whereas FB‐193 and FB‐185 had the lowest SI values in both countries. All genotypes were genotyped via single primer enrichment technology, which resulted in 33,165 SNP markers. The association mapping revealed 162 and 31 significant SNPs in seed traits scored in Germany and Egypt, respectively. A set of seven SNPs was associated with more than one seed trait in Germany, whereas only one SNP was associated with two traits in Egypt. No shared markers were found between the two locations for any of the seed traits. These markers represent potential targets for future breeding programs to enhance seed size and understanding of its genetic control in the faba bean.

## Core Ideas

- Environment × genotype interaction significantly affects seed characteristics in faba beans.
- Only FB_227 and FB_231 showed stable performance across locations with high values for seed characteristics.
- Environment × genotype interaction hindered the detection of stable markers for seed characteristics using GWAS.

## Plain Language Summary

This study investigated how different faba bean genotypes grow and perform in different environments. We found that the environment plays an important role in how yield‐related traits like seed size and shape appear. To address this, we used a selection index, a method that combines all seed traits, to help identify the best‐performing genotypes across regions. Using this approach, we identified two promising faba bean lines, FB_227 and FB_231, that could be valuable for future breeding programs aimed at improving seed traits. We also discovered several new genetic markers linked to important seed traits. Some of these markers affect multiple seed traits at the same time, making them especially useful for marker‐assisted selection

**Abbreviations**
AACs: ADP/ATP carrier proteins
ABA: abscisic acid
DTX49: detoxification 49 proteins
GEI: genotype‒environment interaction
GWAS: genome‐wide association study
LD: linkage disequilibrium
MET: multi‐environment trial
NFD2: nuclear fusion defective 2
NUP1: nuclear pore complex protein
PCA: principal component analysis
*Q*–*Q* plot: quantile‒quantile plot
QTL: quantitative trait loci
SI: selection index
SNP: single‐nucleotide polymorphism
SPET: single primer enrichment technology

## 1 INTRODUCTION

Faba bean (*Vicia faba* L.) belongs to the Leguminosae family. The main advantages of legumes are their biological benefits, such as the fixation of atmospheric nitrogen due to a symbiotic relationship with soil bacteria known as rhizobia (Gutierrez et al., 2024). Faba bean (*V. faba* L.) has one of the highest yield potentials and protein contents (average ∼29%), carbohydrate contents (58.3%), and dietary fiber content (25.0%) among the grain legumes (Dhull et al., 2022). In most cases, faba beans grow well in cool and moist conditions typical of temperate climates (Sallam, Amro, et al., 2024). Many people in the Middle East and African countries use faba beans to prepare diverse local dishes for breakfast, lunch, and dinner. In the agricultural sector, faba beans are essential for crop rotation and nitrogen fixation, with soil values ranging between 50 and 200 kg N ha^−1^ (Gutierrez et al., 2024). However, despite all the previous benefits, total production in Egypt decreased at a rate of about 157,100 tonnes per year because of population growth, and abrupt climate change, creating a widening gap between local production and consumption (Soliman et al., 2024). Therefore, it is necessary to study the diversity of faba beans and their environmental interactions to improve yield and quality by improving the seed characters. Faba bean requires comprehensive data on its seed‐related traits for effective yield and quality improvement (Zhao et al., 2023).

Seed characteristics are significant and directly affect yield and quality. The quality of seeds affects crop yield improvement in the most important crops, such as soybeans and wheat (Tayade et al., 2023). Moreover, multiple seed traits are positively correlated, which helps with screening for better faba bean quality phenotypes. Analyzing the physical characteristics of seeds serves as a preliminary basis for grouping faba bean accessions, guiding germplasm characterization and genetic improvement (Khamassi et al., 2021). The market acceptance of new varieties of faba beans depends on other traits, including seed shape. The evaluation of technological characteristics is an important aspect of selecting and recommending new varieties to increase farmer and consumer acceptance (A. A. Ahmed, Mohamed, et al., 2022). Seed size plays a major role in determining yield potential in other grain legumes such as adzuki beans, indicating that breeders can select high‐yielding cultivars based on seed size (Hu et al., 2022). Faba bean yield is highly unreliable due to a significant level of genotype–environment interaction (GEI) (Cernay et al., 2016). Previous studies have reported considerable variation in genotype × environment (G × E) interactions in faba bean (Haile & Banteayehu, 2025; Skovbjerg et al., 2020). Therefore, it is crucial to use stable genotypes that are well‐adapted to a wide range of environments (Papastylianou et al., 2021). By studying cultivars and genotypes in multi‐environment trials (METs), breeders and agronomists can detect and comprehend GEIs for the best‐performing genotypes in distinct environments (Sharifi et al., 2017). Conducting experiments in METs is important for understanding and analyzing GEIs, enabling the identification of stable and widely adapted genotypes that can be recommended for production in target environments (Temesgen et al., 2015). The phenotypic and quality traits of faba bean seeds are easily affected by the environment (Backouchi et al., 2015).

Genetically, the faba bean has a diploid chromosome number of 2*n* = 2*x* = 12. It is an autogamous plant with partial outcrossing ranging from 20% to 80%, with a 13,000 Mb genome size (Johnston et al., 1999). As a result, the high percentage of faba beans outcrossing hinders a little bit the genetic improvement of faba beans through breeding programs. Owing to the lack of effective solutions for transgenic technologies for faba beans, the use of existing genetic varieties is a major component of a breeding program. Therefore, the use of molecular markers is very effective in understanding genetic diversity and seed traits and might be helpful for breeders (Sallam, Amro, et al., 2024). Genome‐wide association studies (GWASs) are now important tools for identifying markers strongly associated with seed traits. Genotypic and phenotypic data are required to understand the genetic basis of natural variation and complex traits. GWAS can be used to putatively identify genomic regions associated with target traits, and then it should be used in a further functional and validation study (Torkamaneh & Belzile, 2022). Recently, a few studies have investigated genetic variation in seed characteristics and focused on identifying molecular markers associated with seed traits. These studies utilized reference genomes to pinpoint markers through various methods, including the 90K single primer enrichment technology (SPET) genotyping assay (Jayakodi et al., 2023), 21,345 single‐nucleotide polymorphism (SNP) array (Zhao et al., 2023), and the 130K SNP chip developed from RNA‐seq data (Baker, 2020; Ohm et al., 2024). However, only a few GWAS studies on faba beans have used diverse panels to date (Ohm et al., 2024; Lippolis et al., 2025). Therefore, more research efforts are needed to identify the genetic control of seed traits in different faba bean populations.

The objectives of this study were to (1) investigate the genetic variation in seed traits in a highly diverse faba bean population and (2) identify SNPs associated with seed characteristics via a GWAS.

## 2 MATERIALS AND METHODS

### 2.1 Plant material

The diversity consisted of 110 faba bean genotypes that originated in Egypt. These genotypes were collected from the following institutes:

1. Leibniz Institute of Plant Genetics and Crop Research (IPK): Note that 99 bean genotypes were collected from the Department of Gene Bank, IPK, Germany.
2. Egyptian universities and research institutes (EURIs): Note that 11 genotypes were collected from different breeding programs established at some Egyptian universities and research institutes.

The list of genotypes used in this study is provided in Table S1. Self‐propagation was performed for all the genotypes for 3 years in isolated cages at the Experimental Field Station, Department of Genetics, Assiut University.

**Core Ideas**

- Environment × genotype interaction significantly affects seed characteristics in faba beans.
- Only FB_227 and FB_231 showed stable performance across locations with high values for seed characteristics.
- Environment × genotype interaction hindered the detection of stable markers for seed characteristics using GWAS.

### 2.2 Experimental layout and seed traits phenotyping

The genotypes were grown at two locations in October 2023 at the experimental field station, Department of Genetics, Assiut University, Egypt (27°11′20.36″ N, 31°10′06.45″ E), and the Experimental Field Station at the Leibniz Institute of Plant Genetics and Crop Plant Research (IPK), Germany (51°49′25.0″ N, 11°17′34.8″ E). Climatic data (average temperature and humidity) for the two locations were recorded daily to estimate the average monthly trends for temperature and humidity. (Figure S1).

In Egypt, a randomized complete block design with three replications (*R* = 3) was employed, and 10 seeds were handily sown in 1.5‐m rows (one row/genotype), with a spacing of 10 cm between seeds and 50 cm between rows. In Germany, the genotypes were sown in plots (two rows in total, 1 m × 1.5 m) using a completely randomized block design with three replications for each genotype. After being harvested, the seeds of all the genotypes were phenotyped for their seed traits in the Plant Genetics Lab, Faculty of Agriculture, Assiut University.

From each replication, a set of 15 seeds was collected, and the following traits were measured via ImageJ software: seed length (SL) (cm), seed width (SW) (cm), and seed area (SA) (cm^2^) (Schneider et al., 2012). Moreover, the 1000‐seed weight (TSW) was scored by counting and weighing 50 seeds. Then the weight of 50 seeds was multiplied by 20.

### 2.3 Statistical analysis of the phenotypic data

Analysis of variance (ANOVA) and broad‐sense heritability were performed for all the traits via PLABSTAT software (Utz, 1997). All the graphics of the morphological traits were plotted via the SR plot website.

The statistical model used the following model to analyze the morphological characteristics scored:

$$Y_{ijk}=\mu +g_{i}+r_{j}+l_{k}+{\mathrm{lg}}_{ki}+\mathrm{lg}r_{ijk}\left(\mathrm{error}\right).$$

where *Y_ijk_* is the observation of genotype *i* in replication *j* at location *k*, *μ* is the general mean, and *g_i_*, *r_j_*, and *l_k_* are the main effects of genotypes, replications, and locations, respectively. lg*_ki_* is the genotype × location (G × L) interaction, and lgr*_ijk_* is the interaction between genotype *i*, replication *j*, and location *k*. Genotypes and locations were considered fixed effects, whereas replications were considered random effects. Broad‐sense heritability (*H* ^2^) estimates for each trait were calculated from genotypic variance (*σ* ^2^ *G*)/phenotypic variance (*σ* ^2^ *p*) via the HERTI command in PLABSATA. The variance components were estimated by PALBSTAT using the GENOT/1 command. The percentage of variance explained by each source (V.cp%) was calculated manually according to the following formula used by Carena et al. (2010):

$$V.\mathrm{cp}\%=\frac{\mathrm{Var}.cp_{\mathrm{source}}}{\mathrm{Var}.cp_{\mathrm{sources}}}\times 100$$

where Var. cp_source_ is the variance component of the corresponding source of variation, and ∑Var. cp_sources_ is the sum of all variance components.

The Spearman rank correlation coefficient was attributed to the phenotypic correlation between traits.

### 2.4 Multi‐environment testing

The multivariate stability analysis was performed graphically based on the GGE (genotype + genotype × environment) biplot to explain the G × E interaction, via R software using the multi‐environment trial analysis (METAN) package (Olivoto & Lúcio, 2020). The tested locations were analyzed without scaling (“Scaling = 0”) to generate tester‐centered (“Centering = 2”) GGE biplots (Singh et al., 2020).

### 2.5 Selection index

The optimum selection index (SI) at each location was calculated to improve seed characteristics. The SI was used to improve TSW (*X*_1_) via three auxiliary traits: SA (*X*_2_), SW (*X*_3_), and SL (*X*_4_) as follows:

$$\mathrm{SI}=b_{1}X_{1}+b_{2}X_{2}+b_{3}X_{3}+b_{4}X_{4}$$

The Smith–Hazel index coefficient **b** vector was calculated as described by Baker et al. (2020) as **b** = **P** ^**−1**^ **G**, where **P** ^**−1**^ is the inverse of the phenotypic variance‐covariance matrix for the traits; **G** is a matrix that includes the estimates of genotype and covariance, which are *b*_1 _= 0.8944, *b*_2 _= 2.3979, *b*_3 _= −24.0253, and *b*_4 _= −21.8654, while for traits scored on seeds grown at Assiut University (Egypt), *b*_1 _= 0.8649, *b*_2 _= 0.8298, *b*_3_ = −18.6716, and *b*_4 _= 3.8457, where *b*_1_, *b*_2_, *b*_3_, and *b*_4_ are the index coefficients. We selected the 20 genotypes with the highest and lowest values for the target traits at each location based on the SI and then selected the common genotypes between them.

### 2.6 Genetic analysis

#### 2.6.1 DNA extraction and genotyping

Genomic DNA was extracted from all the genotypes by Trait Genetics. All the genotypes were subsequently genotyped via SPET (Barchi et al., 2019). As a result, a total of ∼50K SNPs were generated. After filtering for a minor allele frequency of 0.05 and removing markers and genotypes with more than 30% missing data, a total of 33,165 SNPs across 103 genotypes remained for further genetic association analysis.

#### 2.6.2 Analysis of population structure

The population structure was analyzed using STRUCTURE 3.4.0 (Falush et al., 2007) software to determine the optimal number of subpopulations that best explained the population structure. The analysis was run with burn‐in periods of 100,000 and 100,000. Markov chain Monte Carlo replications, and the ad hoc statistic was used to determine the correct estimated number of clusters with the Structure Harvester online program. The population number (*K*) was tested from 1 to 10, with 20 iterations for each group. Subpopulations were determined according to a threshold of ≥ 0.70 inferred ancestries. Genotypes with an identity value under this threshold were intermixed.

#### 2.6.3 Genome‐wide association analysis

A GWAS was conducted for each trait via R software via a memory‐efficient, visualization‐enhanced, and parallel accelerated (rMVP) package (Yin et al., 2021). In this study, we tested the marker‐trait associations via a mixed linear model, a general linear model, and a fixed and random model of circulating probability unification (FarmCPU). The kinship (kin), principal component analysis (PCA), and PCA + kin were incorporated into each of the three models, resulting in a total of nine GWAS models. We choose the optimal statistical model for each trait based on the distribution of the expected and observed *p*‐values in the quantile–quantile plot (*Q*–*Q* plot) as recommended by Alqudah et al. (2020). The significant markers associated with the traits studied were identified via *p*‐value ≤ 0.001 (−log10 *> *3.00) and suggestive *p*‐values (Duggal et al., 2008), which were calculated as SP = 1/*N*, where *N* represents the total number of markers used.

Analysis of linkage disequilibrium (LD; *r^2^*) was performed among significant markers located on the same chromosome via TASSEL v.5.2.5 (Bradbury et al., 2007). To identify the putative candidate genes of common SNP markers associated with more than one trait in each location, we used the Hedin faba bean genome published by Jayakodi et al. (2023) to download the sequences of each significant SNP (https://projects.au.dk/fabagenome/genomics‐data). These sequences were then blasted against the *Medicago truncatula* reference genome to identify the corresponding proteins of each gene using BLASTx at NCBI (https://blast.ncbi.nlm.nih.gov/Blast.cgi)

## 3 RESULTS

### 3.1 Genetic variation in seed characteristics in faba bean

**FIGURE 1:**
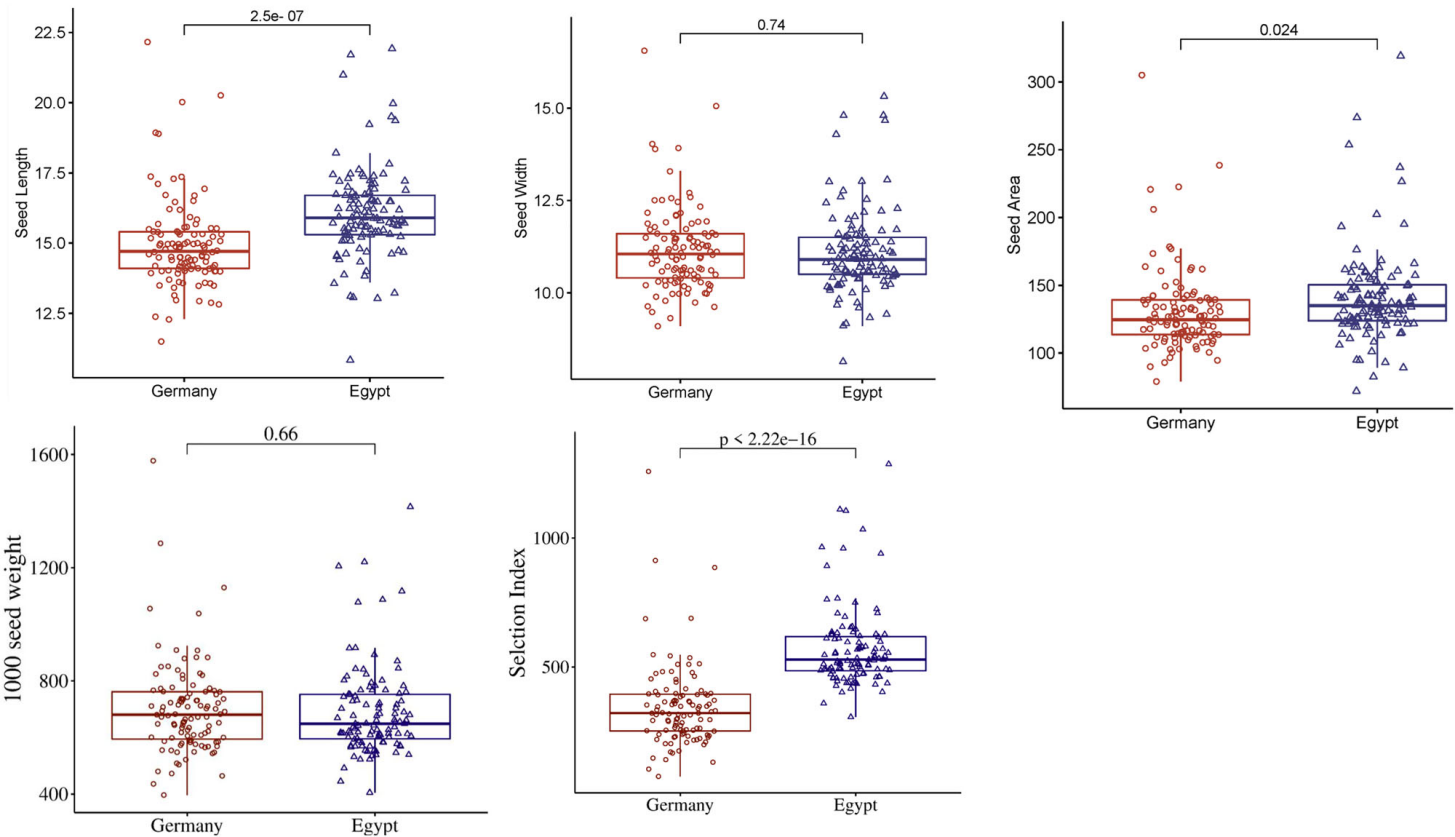
Boxplot shows the minimum, maximum, and mean of five traits for 110 genotypes in two locations.

The results revealed a wide range of variations in the five traits among the genotypes evaluated between the two locations (Figure 1). There were significant differences between the two locations in all traits except TSW and SW. On average, the genotypes in Egypt performed better than those evaluated in Germany for SI, SL, and SA. Genotypes in Egypt have greater values for seed traits (SL and SA) and higher SI values than those evaluated in Germany. SW had the same average in both countries.

**FIGURE 2:**
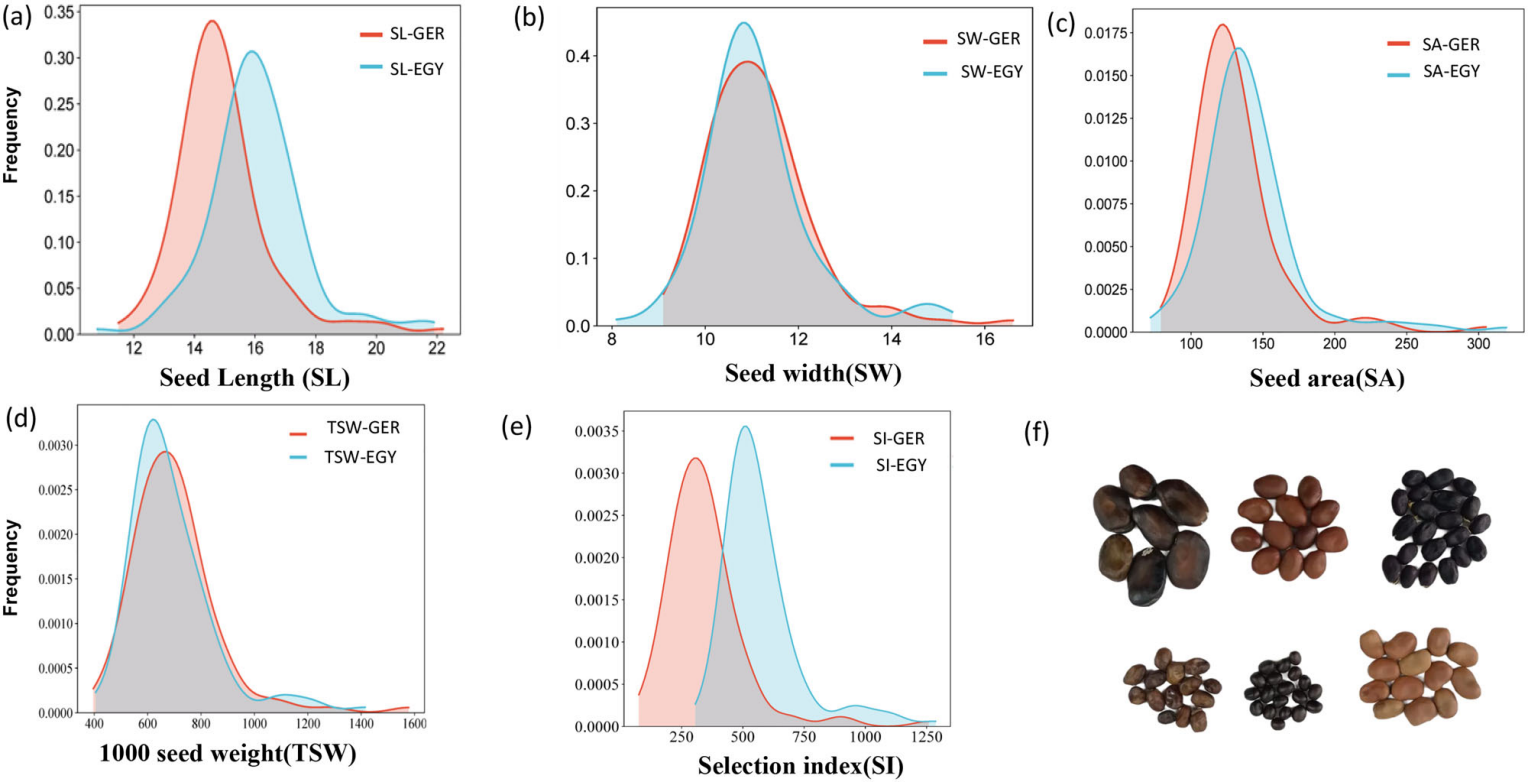
(a–e) Kernel density estimation of five traits between two locations for 110 genotypes. (f) Variation in seed traits among genotypes in the tested population.

The distributions of all the genotypes for each trait at each location are presented in Figure 2. All traits were normally distributed in two locations. The minimum, maximum, and mean values for each trait in each country are presented in Table S2. The results of the ANOVA are presented in Table 1. The ANOVA results revealed a high level of variability among genotypes and locations. The genotypes (G) varied significantly in TSW, SA, SW, and SL. Significant differences were recorded among the locations (L) for all the traits measured except SW. The G × L interactions highly significantly differed for all the traits and exhibited high variance contributions for all traits studied, ranging from 39.5% for SL to 49.9% for TSW. The heritability (*H* ^2^) ranged from 0.92 for SW to 0.96 for TSW. The results of the ANOVA for seed traits scored in Egypt and Germany are presented in Tables S3 and S4, respectively. The results revealed highly significant variation among the genotypes for all the traits in each country.

**TABLE 1:** Analysis of variance for all traits scoring of 110 genotypes through the two locations, Egypt and Germany.

Source of variation	*df*	TSW			SL			SW			SA		
		MS	V.cp%	*F*	MS	V.cp%	*F*	MS	V.cp%	*F*	MS	V.cp%	*F*
L	1	16,177.293	1.8	81.99**	204.6	13.5	287.07**	1.047	0.1	2.78+	16,177.2	2.9	81.99**
R	2	2169.639	0.00	11.00**	10.86	1.01	15.25**	4.229	0.8	11.23**	2169.6	0.57	11.00**
G	109	3940.385	40.2	19.97**	8.99	30.2	12.62**	4.761	36.5	12.64**	3940.3	37.77	19.97**
G × L		2516.0087	49.9	12.75**	6.12	39.5	8.60**	3.08	45.1	8.19**	2516.0	46.80	12.75**
Error		197.305	7.9		0.71	15.6		0.376	18.8		197.3	11.9	
*H* ^2^		0.96			0.92			0.92			0.94		

Abbreviations: *df*, degree of freedom; G, genotype; *H* ^2^, broad‐sense heritability; L, location; MS, mean square; R, replication; SA, seed area; SL, seed length; SW, seed width; TSW, 1000‐seed weight; V.cp%, variance component percentage.

** Significant at the 0.01 level of the probability.

**FIGURE 3:**
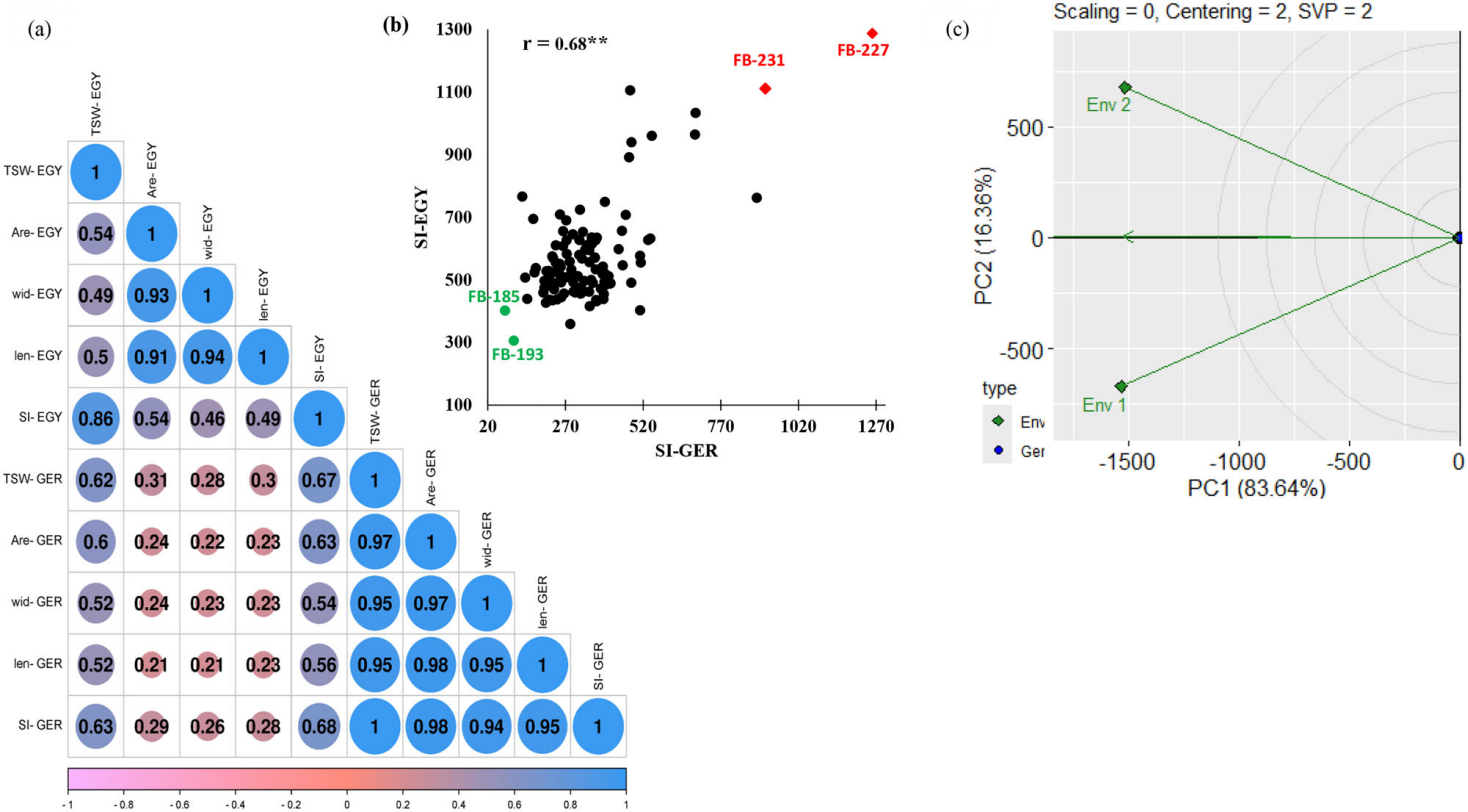
(a) Person correlation matrix of five traits for 110 genotypes between the two locations. (b) Scatter plot of the selection index between the two locations. Red genotypes exhibit a high selection index value, while green genotypes display a low selection index value. (c) GGE biplot based on selection index (SI) values of 110 genotypes across two environments, Env 1 refers to Germany and Env 2 refers to Egypt.

The correlation coefficients among the seed characteristics at the two locations are presented in Figure 3a. The results revealed highly positive correlations among all the traits in Egypt and Germany. The correlation between TSW and seed traits in Germany was greater than that between TSW and seed traits in Egypt. The highest significant correlation was between SW and SL in Egypt (*r* = 0.94**), whereas SA and SL had the highest positive significant correlation in Germany (*r* = 0.98**). The seed index was positively correlated with all the seed traits in Germany and Egypt. Interestingly, a lowly significant correlation was found between the seed traits of the two countries. However, the SI in Germany had a highly significant positive correlation with the SI in Egypt (*r* = 0.68**) (Figure 3b). Moreover, the SI in Egypt was significantly positively correlated with all the seed traits harvested in Germany, whereas a significantly weak correlation was found between the SI in Germany and the seed traits in Egypt.

Using SI values, the performance of the two environments was evaluated using the GGE model based on the environment‐focused relationships between the environment biplot (Figure 3c). Here, PC1 and PC2 accounted for 83.64% and 16.36% of the total variance, respectively. Environments that form obtuse angles have a negative correlation, and the strength of the relationship is determined based on the size of the angles. There is a wide angle between Env1 and Env2, indicating a crossover G × E interaction. The single arrowhead line in the biplot indicated the average environment (Figure 3c). Genotypes differed clearly on PC2 across environments (Env 1 and Env 2), but showed nearly identical coordinates on PC1, which accounted for most of the total variation. Based on genotype performance, the slopes of the genotypes in the two environments indicated the presence of all types of G × E interactions (Figure S2).

**FIGURE 4:**
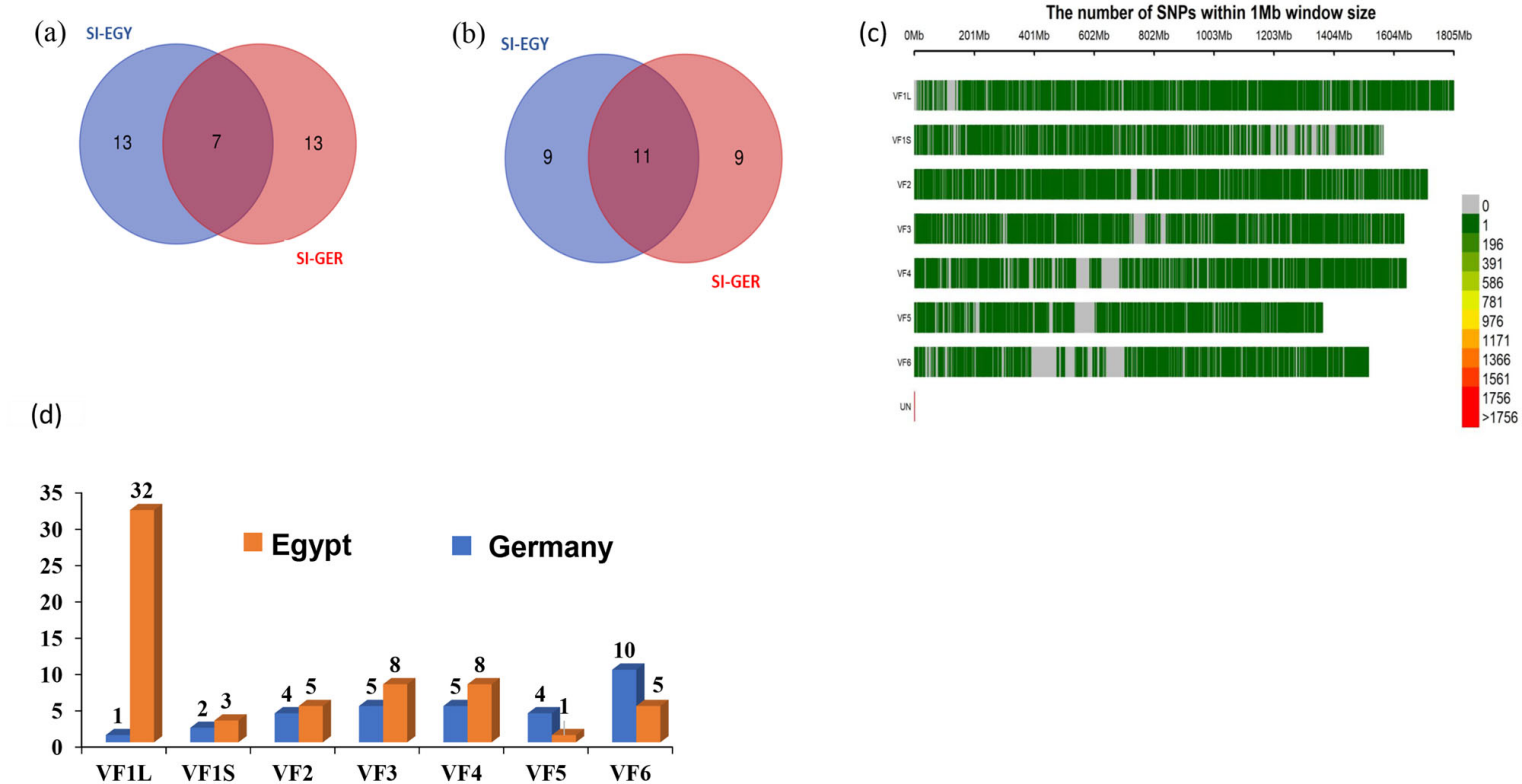
(a)Venn tool for the top 20 genotypes of selection index between the two locations. (b) Venn tool for the lowest 20 genotypes of selection index between the two locations. (c) The distribution of single‐nucleotide polymorphism (SNP) makers across all the faba bean chromosomes in each location. (d) The distribution of significant SNPs on each chromosome in each location.

Based on the SI in both countries, we selected the highest and the lowest 20 genotypes in each location for the selected traits. As a result, we found that seven genotypes were among the top 20 genotypes in both countries (Figure 4a; Table S5), and 11 genotypes were among the lowest 20 genotypes for SI in both countries (Figure 4b; Table S5). FB‐231 and FB‐227 performed very well in both countries, as they had the highest SI values in both countries, whereas FB‐193 and FB‐185 had the lowest SI values in both countries (Figure 3b).

### 3.2 GWAS of seed characteristics in faba beans

SPET genotyping generated a set of 33,165 SNP markers that were distributed on the six faba bean chromosomes (Figure 4c). Chr 1 L had the highest number of SNP markers, whereas Chr 5 had the lowest number of SNP markers (Figure 4d).

**FIGURE 5:**
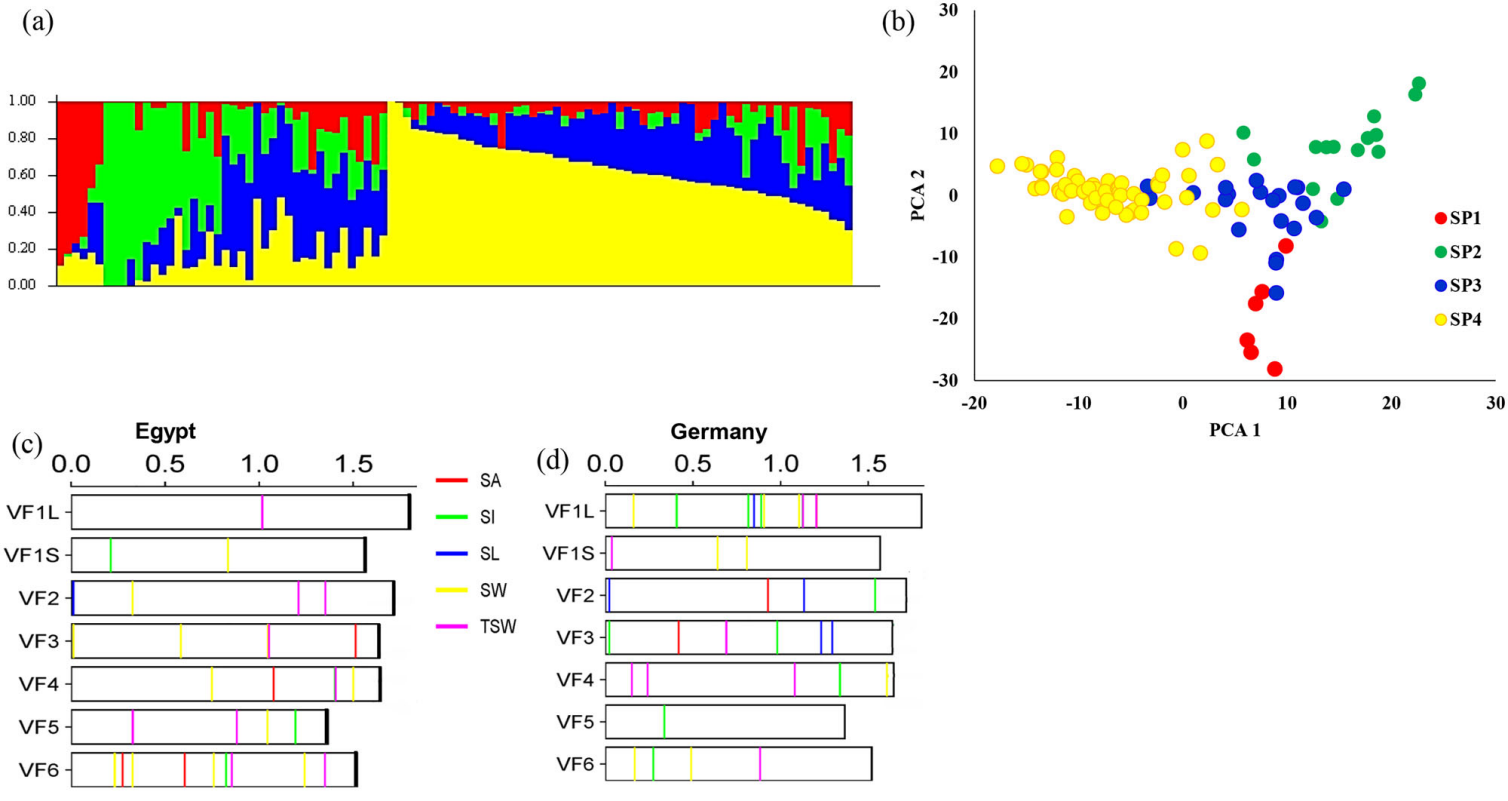
(a) Analysis of population structure for the 102 faba bean genotypes. (b) Principal component analysis for the 102 faba bean genotypes (grouping was based on the results of population structure). (c) and (d) The distribution of the significant markers on the faba bean chromosome in Egypt and Germany, respectively.

The analysis of population structure is presented in Figure 5a. The STRUCTURE analysis divided the Egyptian panel into four subpopulations, indicating the presence of population structure in this panel. Also, the results of PCA based on SNP markers for all genotypes are presented in Figure 5b. A GWAS was performed for all seed traits in both countries via 33,165 SNP markers and all seed traits from both countries. The significant markers were detected at two thresholds: a suggestive *p*‐value of 3.01 × 10^−5^ (Table S6) and *p *< 0.001 (Table S7). In this study, we focused on significant markers detected at the suggestive *p*‐value. A summary of the GWAS results for all the seed traits is presented in Table 2. At the suggestive *p*‐value, the total number of significant SNPs detected in Germany (47) was greater than that detected in Egypt (31). SW in Egypt had the greatest number of significant SNPs (12), whereas in Germany, SI had the greatest number of significant SNPs (13). The distribution of the significant markers on the faba bean chromosome is presented in Figure 5c,d for Egypt and Germany, respectively.

**TABLE 2:** Summary of significant single‐nucleotide polymorphism (SNP) markers associated with seed traits in each location at a suggestive *p*‐value.

Trait	Location	Model	No. of significant SNPs	*p*‐value range	Allele effect	*R* ^2^ (%)	No. of minor SNPs	No. of major SNPs
SA	EGY	FarmCPU + PCA	5	5.42E‐13–0.00000404	−11.08 to 18.886	4.454–33.276	2	3
	GER	PCA FarmCPU	9	3.04E‐18−0.0000148	−14.68 to 15.124	2.753–15.99	4	5
	Total		14					
SW	EGY	PCA FarmCPU	12	1.06E‐08–0.0000237	−0.514 to 0.6552	1.92–31.83	4	8
	GER	Kin + FarmCPU	9	2.71E‐08– 0.0000306	−0.44058 to 0.454848	3.705–15.497	4	5
	Total		21					
SL	EGY	MLM	1	3.17E‐05	(−1.7)	28.317	0	1
	GER	PCA FarmCPU	8	1.8E‐13–0.0000017	−0.905709 to 0.8359904	5.71–21.463	3	5
	Total		9					
TSW	EGY	PCA FarmCPU	9	1.38E‐11–0.0007167	−80.41 to 69.520	3.455–28.93	5	4
	GER	PCA FarmCPU	8	6.27E‐16–0.0000264	−77.50764 to 76.690884	5.048–18.665	3	5
	Total		17					
SI	EGY	Kin + FarmCPU	4	0.0000001–0.0000037	−54.47 to 58.228	3.17–19.84	3	1
	GER	PCA FarmCPU	13	2.05E‐12–0.0009	−64.4 to 89.7	0.001–19.8	9	4
	Total		17					

*Note*: *R* ^2^ refers to the phenotypic variation explained by the SNP marker (%). Major SNPs indicate that these SNPs explained more than 10% (*R* ^2 ^> 10%), while minor SNPs indicate that these SNPs explained less than 10%(*R* ^2 ^<10%).

Abbreviations: EGY, Egypt; FarmCPU, fixed and random model of circulating probability unification; GER, Germany; Kin, kinship; PCA, Principal component analysis; SA, seed area; SI, selection index; SL, seed length; SW, seed width; TSW, 1000‐seed weight.

**FIGURE 6:**
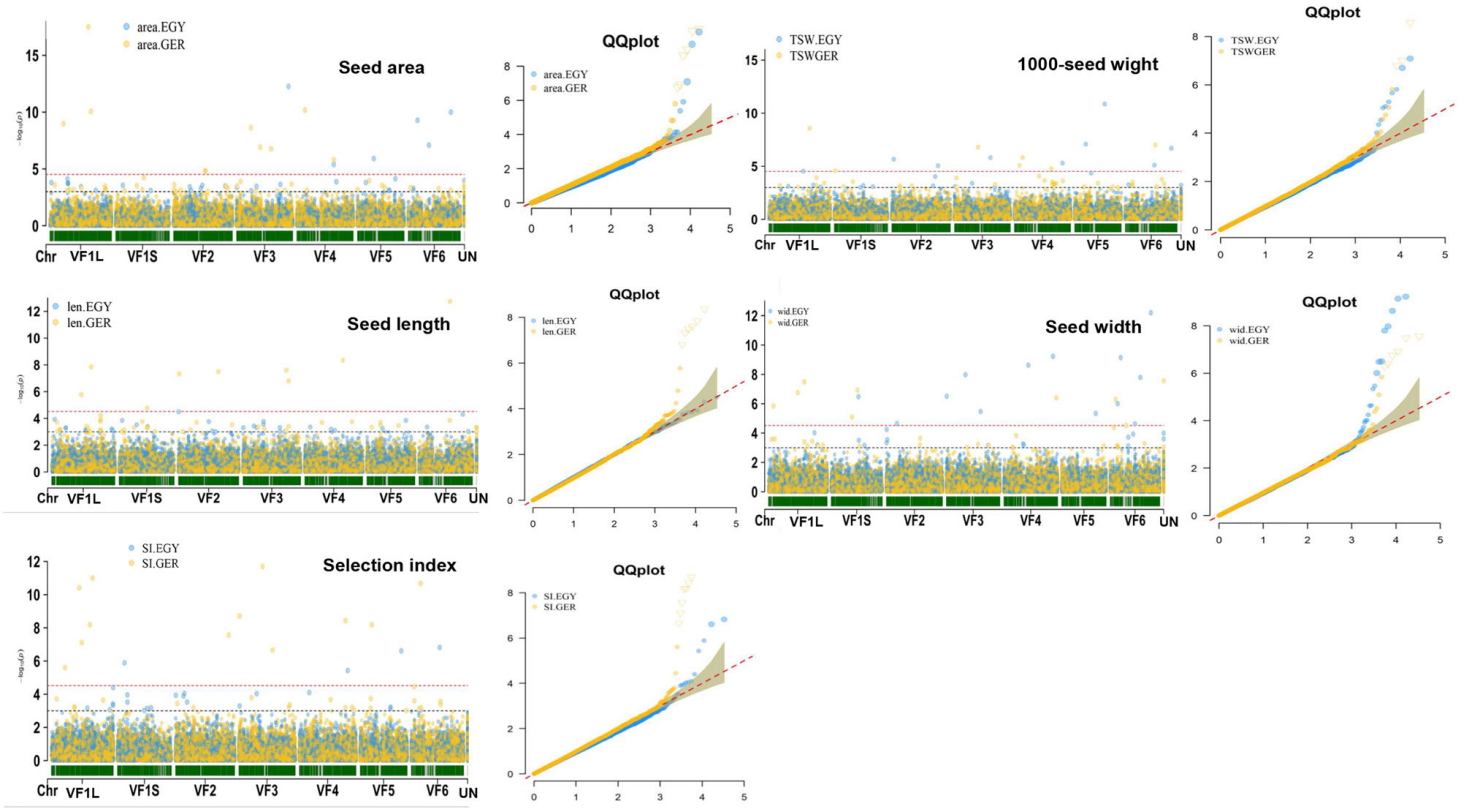
Manhattan plots and the quantile‒quantile plot (*Q*–*Q*) plots from genome‐wide association study (GWAS) results. Egypt is identified by blue single‐nucleotide polymorphism (SNP) markers and Germany is identified by yellow SNP markers. The upper dotted lines indicate a suggestive *p*‐value, while the lower lines indicate 0.001 *p*‐value.

Based on the results of the *Q*–*Q* plot, the FarmCPU GWAS model was the best fit, with all traits scored in both countries except for SI in Egypt and SW in Germany. The results of the Manhattan plot and *Q*–*Q* plot for each trait are presented in Figure 6. The phenotypic variation explained by the marker (*R* ^2^) is presented in Table 2. Interestingly, the number of SNPs with major effects (*R* ^2 ^> 10%) was greater than that with minor effects for all seed traits in both countries except for SI in Germany and TSW in Egypt.

No significant markers were shared between the two locations for any trait. However, we found markers associated with more than one trait in each location (Table 3). A set of seven SNPs was found to be associated with more than one seed trait in Germany, whereas only one SNP was associated with two traits (SA and SW) in Egypt. Among the eight significant SNPs detected for seed traits in Germany, three were associated with three traits, whereas the remaining markers were associated with two traits. Notably, most of the common markers (four markers) were found to be associated with TSW and SA.

**TABLE 3:** The list of common single‐nucleotide polymorphisms (SNPs) markers associated with more than one trait in each location.

Marker	Country	Chro.	Pos.	TSW	SL	SA	SW	SI	Candidate gene	Protein coding
S3_690637753	Germany	Chr3	690637753	×		×		×	R1.3g110280	Nuclear pore complex protein nup1 isoform x1
S1L_1202495575	Germany	Chr1L	1202495575	×				×	R1.1g384840	Protein detoxification4
S1L_406260950	Germany	Chr1L	406260950			×		×	R1.1g256480	Abscisic acid and environmental stress‐inducible protein
S6_882692345	Germany	Chr6	882692345	×	×				R1.6g105680	Anthocyanidin reductase
S4_241701587	Germany	Chr4	241701587	×		×			R1.4g042840	ADP/ATP carrier protein
S4_1080647920	Germany	Chr4	1080647920	×	×	×			R1.4g157640	Protein nuclear fusion defective 2
S1L_1127089322	Germany	Chr1L	1127089322	×	×	×			R1.1g372960	Uncharacterized protein loc11430996
S6_1241132666	Egypt	Chr6	1241132666			×	×		R1.6g160280	LRR receptor‐like serine/threonine‐protein kinase At5g63710

*Note*: x indicates that the marker is assoicated with the respective trait.

Abbreviations: Chro., chromosome; Pos., position; SA, seed area; SI, selection index; SL, seed length; SW, seed width; TSW, 1000‐seed weight.

The analysis of LD (*r^2^*) for the significant marker pairs located on the same chromosome is presented in Figure S3. Among all the marker pairs, two SNPs located on Chr 2 were found to be in complete LD. No significant LD was found among the remaining SNP pairs on the same chromosome.

### 3.3 Potential gene annotation

Gene annotation was performed for the significant markers with multiple related traits (Table 3). The results of gene annotation identified eight potential candidate genes. Notably, three significant markers were found to be located within three different genes that encode 49 detoxification proteins. In Germany, the candidate gene *R1.3g110280*, which contains SNPs associated with TSW, SA, and SI, encodes the nuclear pore complex protein (NUP1) isoform X1. The candidate gene *R1.1g372960* harbored SNPs related to TSW, SA, and SL and encodes the uncharacterized protein LOC11430996. The SNP located within the *R1.1g256480* candidate gene, associated with SA and SI, encodes abscisic acid (ABA) and an environmental stress‐inducible protein. Moreover, the SNP associated with TSW, SL, and SA falls into the *R1.4g042840* gene, which encodes an ADP/ATP carrier protein (AAC). The significant SNP located within *R1.6g105680*, associated with TSW and SL, encodes an anthocyanidin reductase protein. Moreover, the S4_1080647920 SNP associated with TSW, SL, and SA falls into the *R1.4g157640* gene, which encodes a protein nuclear fusion that is defective 2. The candidate gene *R1.1g372960* harbored the S1L_1127089322 SNP (TSW, SA, and SL) and encodes the uncharacterized protein LOC11430996. The only SNP found in Egypt was located within the *R1.6g160280* gene, which encodes the LRR receptor‐like serine/threonine‐protein kinase At5g63710.

## 4 DISCUSSION

### 4.1 Genetic variation in the seed characteristics of faba bean

Seed traits in faba beans are crucial for several reasons, encompassing both agronomic and economic aspects. Seed traits such as length, width, area, and weight in faba bean are positively associated with yield, as larger seeds provide more stored energy for seedling vigor and establishment. Zalama and Leilah (2019) reported that a 100‐seed weight above 110 g improved both vigor and yield per plant. Similarly, Zewide and Ademe (2025) found that larger seeds were linked to higher germination, better establishment, and ultimately greater yields. Faba bean has a large and wide variation in seed traits, especially length, width, area, and weight. Therefore, investigating such variation is useful for selecting high‐yielding faba bean genotypes for breeding programs. Few studies have investigated the genetic variation in seed traits in faba beans. All these studies focused on investigating the genetic variation in seed characteristics in one location (Dewangan et al., 2022; Ramadan et al., 2025; Zhao et al., 2023). It is imperative to study the effects of different regions on the stability of genotypes in terms of these important traits. Here, we reported the effects of two completely different locations (Egypt and Germany) on the seed characteristics of a set of 110 Egyptian faba bean genotypes. The analysis of variance revealed highly significant differences in all traits except SW between the two locations. More importantly, all G × L interactions were highly significant for all the traits, indicating that most of the genotypes responded differently across the two locations. High broad heritability estimates were found for the characteristics of the seed. These results indicate that selection for improving seed characteristics is feasible in future faba bean breeding programs. High heritability estimates were previously reported by Skovbjerg et al. (2023) and Ohm et al. (2024) for the same seed traits scored in this study, with *H* ^2 ^> 0.96, which was higher than those reported in a single location or across two locations. Understanding the heritability of seed characteristics in faba beans is crucial for developing effective breeding programs to improve these traits. By focusing on traits with high heritability, breeders can achieve more rapid and predictable gains in seed quality and yield. Interestingly, the combined ANOVA also revealed high heritability estimates, indicating strong genetic control of these traits.

High phenotypic correlations among seed traits in each country indicate that improving more than one trait during selection is feasible. The same high phenotypic variation was previously reported among seed traits in a MAGIC faba bean population, with *r* > 0.88 (Du et al., 2009; Skovbjerg et al., 2023). However, a weakly significant correlation was found among the seed traits between the two countries. This weak correlation could be due to the effect of location, as the countries have very different climate conditions, soil types, altitudes, and agricultural practices. The location where seeds are grown significantly impacts their characteristics due to a combination of environmental, genetic, and management factors (Du et al., 2009). Adapting seeds to specific local conditions or selecting seeds from appropriate regions can lead to better crop performance and sustainability in agriculture. A SI including all the seed characteristics was calculated for each country to better select the high‐performance genotypes for the seed traits. The SI was highly correlated with all seed traits in each country. Interestingly, SI_GER was highly correlated with SI_EGY. Therefore, selecting genotypes with superior seed‐related traits across different environments could be achieved via the SI. The results of genotype selection based on SI revealed seven genotypes with high‐yield traits. Although the significant G × E interaction hinders selection for genotypes across different locations, the SI revealed that FB_227 and FB_231 had stable performance for seed characteristics in both countries, with approximate values. This finding indicates the usefulness of using the SI in breeding programs to identify stable genotypes. The SI has also been widely used to improve frost tolerance in faba beans (Sallam & Martsch, 2015) and other abiotic stress tolerances in different crops (A. A. M. Ahmed et al., 2021; A. A. M. Ahmed, Dawood, et al., 2022). The most important advantage of selection is that it includes a group of traits that promise fruitful results in improving traits in breeding programs for faba bean (Sallam, Dhanapal, et al., 2016) and other crops (Abou‐Zeid & Mourad, 2021; Sallam, Awadalla, et al., 2024).

The 110 Egyptian faba bean genotypes presented considerable genetic variation in terms of seed characteristics and can be successfully used to improve seed yield.

### 4.2 GWAS for seed characteristics

The seed characteristics in faba bean are polygenic traits controlled by many genes. Although some studies reported important markers related to seed traits in faba beans, most of these studies used the traditional quantitative trait loci (QTL) mapping approach (Aguilar‐Benitez et al., 2025; Zhao et al., 2023). GWASs are considered powerful methods for identifying alleles and candidate genes associated with target traits. Compared with traditional QTL mapping, it has more advantages in dissecting the genetic control (Alqudah et al., 2020; Ohm et al., 2024; Skovbjerg et al., 2023). Only two studies have identified allele and candidate genes associated with SW, SA, SL, and seed weight in faba beans via GWAS (Ohm et al., 2024;Skovbjerg et al., 2023). These two studies reported important SNP markers associated with different seed traits in a single location. Therefore, the genetic control of seed traits via GWAS has been poorly investigated. In this study, the effects of the G × E interaction on the genetic control of seed traits were investigated in two different countries, and genetic control in each individual country was reported.

The genetic diversity among genotypes in this population was extensively studied by Sallam, Amro, et al. (2024), who reported high genetic diversity among Egyptian faba bean genotypes. This high genetic diversity is the main reason for the considerable and high genetic variation among genotypes in seed traits found in this population. The analysis of the population structure divided the Egyptian panel into four subpopulations. Investigating population structure is an essential step for GWAS analysis, as it can lead to spurious associations. Therefore, a correct GWAS model should be used to account for population structure. The GWAS in this study was conducted via nine different models, and both PCA and kin analysis were included to correct for the effects of population structure. Moreover, a GWAS was performed on a set of 110 faba bean genotypes. For association mapping, a minimum of 100–500 individuals are required (Ghazy et al., 2024; Kumar et al., 2012; Mourad et al., 2022). Association analysis was performed between a total of 33,117 SNP markers generated from SPET and all seed traits scored in this study. The SPET method was previously used to generate SNPs that were used to identify alleles associated with yield and seed traits in faba beans (Jayakodi et al., 2023; Ohm et al., 2024; Skovbjerg et al., 2023).

In this study, 31 and 47 significant SNPs were detected for seed traits at suggestive *p*‐values in Egypt and Germany, respectively. The *p*‐value < 0.001 was widely used in GWAS studies as a significance threshold (cutoff) (e.g., Javid et al., 2025; Ma et al., 2022; Sallam, Dawood, et al., 2024). Using stringent *p*‐values, such as those derived from the Bonferroni correction or false discovery rate, may result in the loss of important markers/genes with minor effects (Duggal et al., 2008; Kaler & Purcell, 2019). A *p*‐value of 0.001 in GWAS serves as a relaxed yet reasonable threshold for identifying potential candidate associations (Chen et al., 2021). However, functional validation remains critical to confirm the true association. For example, Sallam, Dawood, et al. (2024) validated one important SNP marker associated with leaf wilting at a threshold of *p*‐value < 0.001 in spring and winter populations at the seedling stage. In this study, the number of significant SNPs varied by country, with no overlap across environments, likely due to G × E interactions and differences in phenotypic variation. These interactions can lead to different QTLs being significant in each environment. In our study, SW had the highest significant markers at suggestive *p*‐values in both countries. The highest number of significant markers detected by Ohm et al. (2024) was found for seed size, with nine significant markers, whereas Skovbjerg et al. (2023) obtained similar results by identifying 36 significant SNPs for SW, the greatest number of SNPs compared with other traits. These results confirmed the importance of the SW trait in faba bean, suggesting that it may be controlled by a greater number of genes than other seed traits.

Most of the significant markers detected in this study had *R* ^2^ values > 10%, indicating that these markers have major effects on seed traits. In Egypt, SW had the greatest number of SNPs with major effects, whereas all seed traits had five major SNPs in Germany.

Interestingly, common markers associated with more than one trait were reported in this study in each country. Therefore, these markers could be useful for further validation for use in marker‐assisted selection in specific countries. However, these markers should be validated in different genetic backgrounds or/and locations or/and years (Hashem et al., 2023; Mourad et al., 2018; Sallam, Arbaoui, et al., 2016; Sallam et al., 2023). The lack of common markers for each trait between the two counties could be due to the interaction effects between location and genotype, where marker effects can vary substantially across environments, especially quantitative traits such as TGW (Yan & Kang, 2002). Moreover, the detection of shared markers may be affected by factors such as sample size, statistical power, and experimental error, given the sensitivity of GWAS to these parameters (Korte & Farlow, 2013). The location × genotype interaction hinders the detection of common markers (Eltaher et al., 2021). A GWAS was performed for seed traits scored in different locations and years in Denmark. They reported stable significant markers for SW, SL, SA, and TGW (Skovbjerg et al., 2023). Therefore, stable markers for seed traits may be detectable in different locations or/and early within country locations.

The highest number of significant SNPs found in this study was located on Chr 1 (33 SNPs). The same results were previously reported by Skovbjerg et al. (2023). The *r^2^* (LD) among the significant markers detected on each chromosome was calculated to identify the inheritance pattern of these markers. Most of the marker pairs located on the same chromosome had no LD, indicating that these SNPs can be considered individual QTLs. Only two makers located in Chr 2 had complete LD, and they tended to be coinherited together. At the population level, this population had very low LD according to Sallam, Amro, et al. (2024).

In this study, we compared the physical positions of significant SNPs with those reported in previous GWAS on seed traits in faba bean(Jayakodi et al., 2023; Ohm et al., 2024; Skovbjerg et al., 2023). No significant SNPs were shared between the earlier studies and the present study, suggesting that the SNPs identified here may represent novel loci.

### 4.3 Potential gene annotation

For candidate genes, we discuss the genes that harbor SNPs associated with more than one trait in each location. The NUP1 isoform X1, which is associated with TSW, SA, and SI, plays an essential role in plants by participating in processes related to environmental conditions and cell growth, potentially affecting crop characteristics such as quality and productivity. This protein acts directly on the regulation of stress adaptation‐related genes involved in the expression of transcripts. Abnormalities in the reproduction of both male and female gametes are due to the lack of the nucleoporin NUP1. Moreover, decreased NUP1 gene expression affects the reproductive growth of plants (Bao et al., 2019; Li & Gu, 2020).

Detoxification 49 protein (DTX49), which contains one SNP associated with TSW and SI, participates in the regulatory processes for plant defense that are currently being developed because of damage caused by environmental stress or alien agents that may attack useful cells. Different kinds of detoxification proteins, such as DTX49, belong to the family of membrane transporter proteins. These proteins help remove toxic substances from cells, thereby protecting plants in environments with heavy metals or toxic compounds. Their success in survival is attributed to these mechanisms. The detoxification of proteins in plants also provides the necessary detoxification, which in turn affects the health of the plant; thus, this method has the potential to produce high‐quality crops in environments with pollution or some type of stress (Li et al., 2022). ABA and environmental stress‐inducible protein, associated with SA and SI in this study, play key roles in helping plants respond to various environmental stresses, such as drought, salinity, and cold. ABA often acts as a signaling molecule, activating pathways that lead to the production of stress‐inducible proteins. Through complex signaling networks, ABA coordinates the physiological and molecular responses that improve a plant's ability to tolerate environmental stresses, making it crucial in plant stress biology. It affects seed dormancy, enabling seeds to withstand unfavorable conditions and germinate only when conditions improve (Tuteja, 2007).

The candidate genes associated with TSW and SA encode AACs, which belong to the mitochondrial carrier family and are characterized by multiple transmembrane domains that facilitate the transport of adenosine diphosphate (ADP) and adenosine triphosphate (ATP) (Clémençon et al., 2013). These proteins play crucial roles in cellular energy metabolism by facilitating the exchange of ADP and ATP across the mitochondrial membrane. The proper functioning of AACs is essential for maintaining energy homeostasis within the cell. Disruption of their activity can lead to impaired energy production, which can affect various cellular processes. The ADP/ATP carrier plays a role in the regulation of metabolic pathways. By controlling the levels of ATP and ADP, these proteins help coordinate the energy demands of the cell with its metabolic activities (Clémençon et al., 2013; Qian et al., 2023). The anthocyanidin reductase protein, encoded by the R1.6g105680 gene (thousand‐kernel weight and SL), is indirectly influenced by the biosynthesis of anthocyanins, which are crucial for coloration and nutrition. It additionally results in the generation of other flavonoids, such as tannins (Brown et al., 2002; Jun et al., 2021), which can be the cause of the taste of seeds and the appearance of plants from pests. Anthocyanins have antioxidant properties, which help protect seeds from oxidative stress during growth periods (Simmonds, 2003). The candidate gene, related to TSW and SA, encodes nuclear fusion defective 2 (NFD2) and plays an important role in plant fertilization and embryonic development (Gacek et al., 2017). NFD2 is thought to contribute to the regulation of genetic processes that affect seed growth. Controlling gene expression can influence traits such as the size, shape, and nutritional content of seeds (Portereiko et al., 2006). Future generations of plants can be affected by changes in or mutations in the NFD2 protein that affect heritable seed traits. This can markedly impact on agricultural characteristics, including productivity and disease resistance. The NFD2 protein could interact with other proteins in genetic networks that play a role in seed development and growth.

Finally, the only candidate gene in Egypt, R1.6g160280, was associated with SA and SW. It encodes LRR receptor‐like serine/threonine‐protein kinase At5g63710, which was discovered in *Arabidopsis thaliana*. LRRs are involved in many signal responses to regulate plant growth and development (Chakraborty et al., 2019) and may play an important role in growth control and developmental signals related to plant hormones such as auxins and cytokinins, which play crucial roles in determining seed traits (Chakraborty et al., 2019). The relationships between the functions of the candidate genes and the seed traits confirmed the success of GWAS in identifying important SNPs that can be converted to kompetitive allele‐specific PCR (KASP) markers to genotype different faba bean genetic backgrounds for validation.

In conclusion, the G × L interaction affected phenotypic selection and hindered the detection of stable markers across the two locations. However, the SI, which includes all seed traits, is a better estimate for effectively selecting promising genotypes with target traits across different regions. FB_227 and FB_231 can be used for future breeding programs. Novel SNP markers associated with seed traits and with major effects were reported in this study. The common markers, associated with multiple traits, can be converted to KASP and validated in different genetic backgrounds to be used for marker‐assisted selection in specific countries.

## AUTHOR CONTRIBUTIONS

**Ahmed Sallam**: Conceptualization; data curation; formal analysis; methodology; resources; supervision; validation; writing—original draft; writing—review and editing. **Salwa M. Mahmoud**: Data curation; formal analysis; methodology; software; validation; visualization; writing—original draft. **Ahmed Amro**: Data curation; resources; supervision; validation; writing—original draft. **Ameer Elfarash**: Investigation; supervision; validation; visualization; writing—original draft. **Andreas Börner**: Resources; supervision; validation; visualization; writing—review and editing.

## CONFLICT OF INTEREST STATEMENT

The authors declare no conflicts of interest.

## Supporting information



Supplementary Material

Supplementary Material

## Data Availability

This published article and its Supporting Information files include all the data generated or analyzed during this study. The sequence data that support the findings of this study are available from the authors, but restrictions apply to the availability of these data, which were used under license from the Academy of Scientific Research and Technology (ASRT) and Assiut University, Egypt, for the current study, and so are not publicly available. Data are, however, available from the authors upon reasonable request and with permission from the Academy of Scientific Research and Technology (ASRT) and Assiut University, Egypt. All other data generated or analyzed during this study are included in this published article and its Supporting Information files.
