# Supplementary material for: Effects of genotype × environment interactions on the morphological and genetic aspects of the seed characteristics of faba beans
Source: Plant Genome. 2025 Oct 15;18(4):e70133. doi: 10.1002/tpg2.70133 (PMC12528823; doi:10.1002/tpg2.70133)
Supplement: Supplementary file 1 — Supplementary Material [file TPG2-18-e70133-s001.pdf]

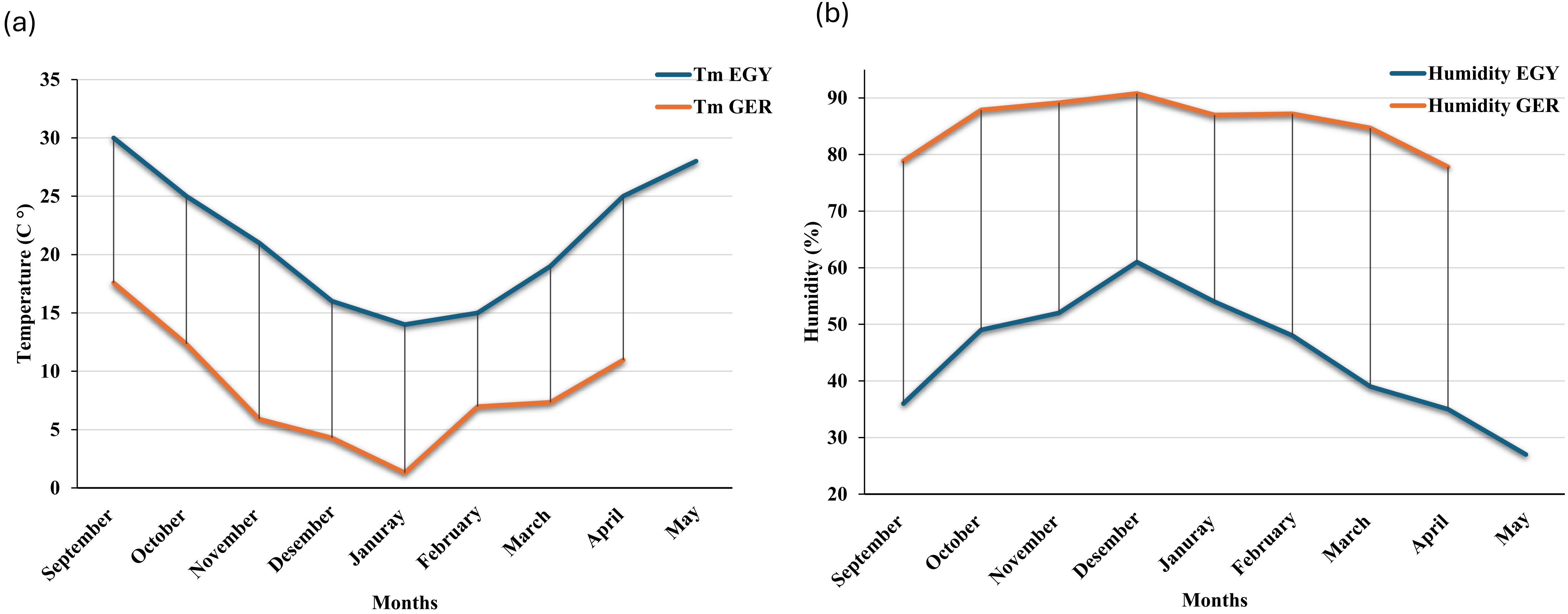

Supplementary Figure 1: Average temperature (a) and humidity (b) in two locations; Egypt (EGY) and Germany (GER)

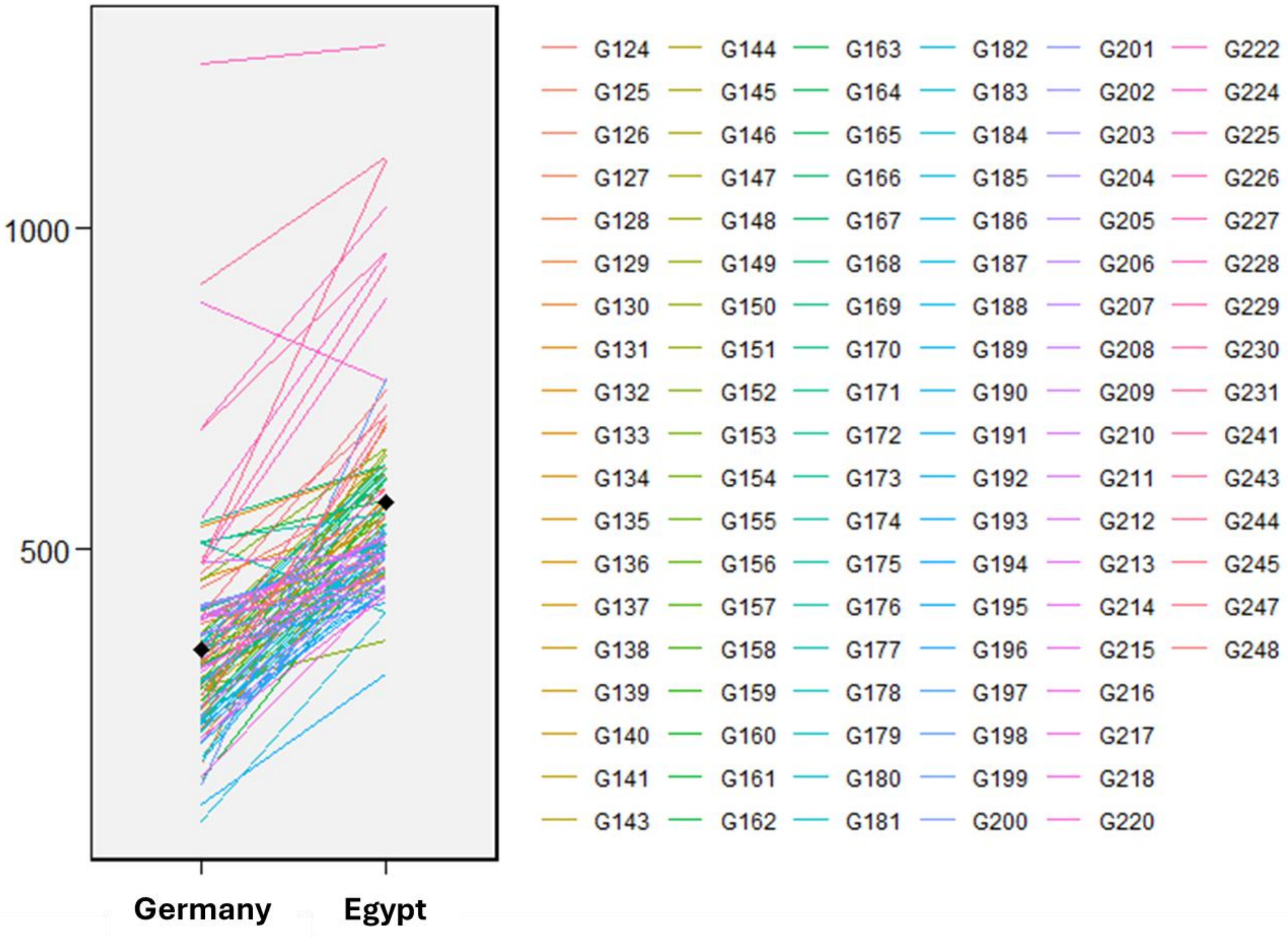

Supplementary Figure 2: The individual performance of 110 genotypes varied greatly over the two environments ENV1 refers to Germany and ENV2 refers to Egypt.

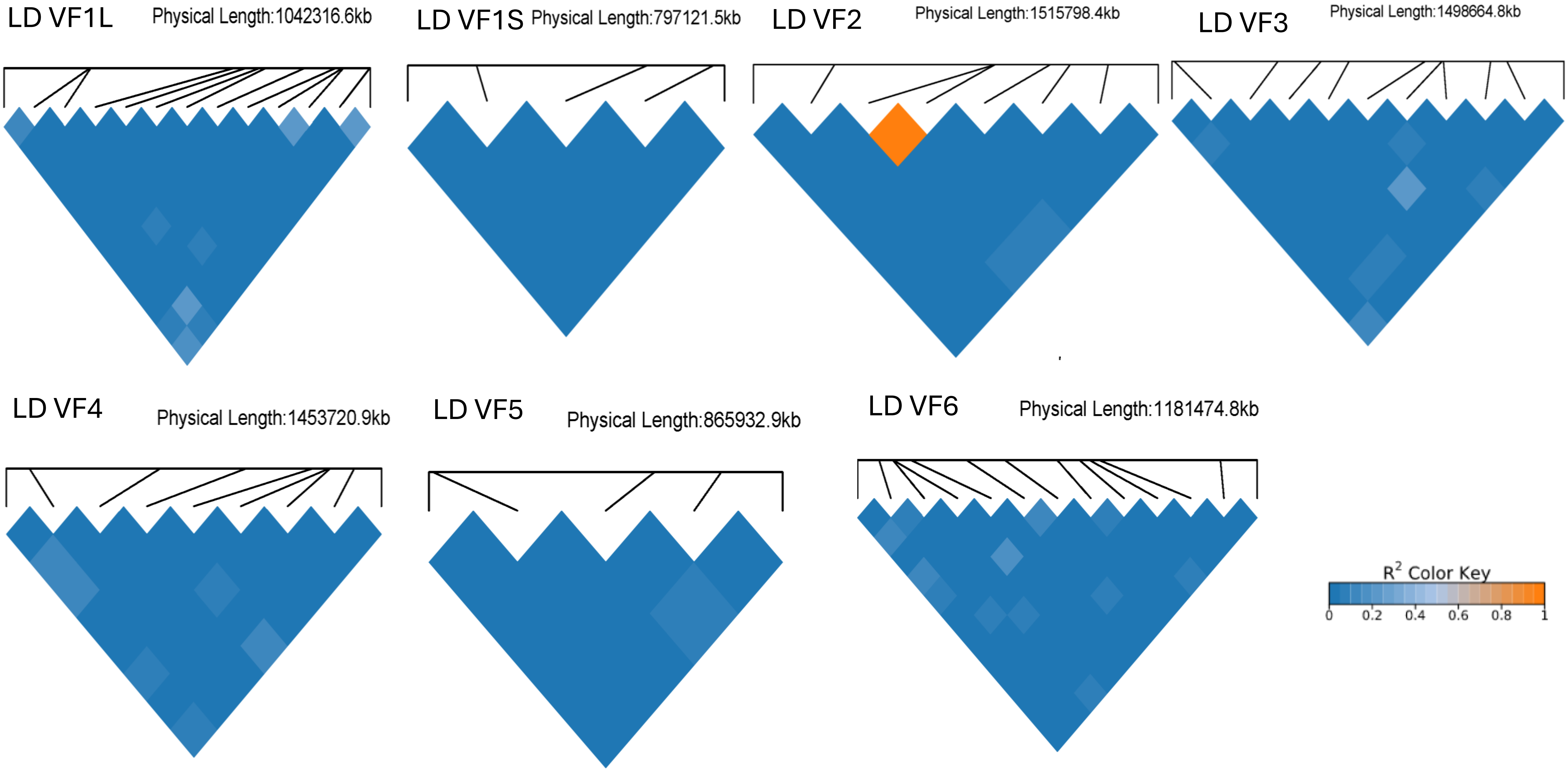

Supplementary Figure 3: A linkage disequilibrium (LD) based heatmap showing LD (r^2) values among SNP markers located on the same chromosome.
